# Differentially penalized regression to predict agronomic traits from metabolites and markers in wheat

**DOI:** 10.1186/s12863-015-0169-0

**Published:** 2015-02-26

**Authors:** Jane Ward, Mariann Rakszegi, Zoltán Bedő, Peter R Shewry, Ian Mackay

**Affiliations:** Plant Biology and Crop Science, Rothamsted Research, Harpenden, AL5 2JQ UK; Agricultural Institute, Centre for Agricultural Research, Hungarian Academy of Sciences, P.O. Box 19. 2462, Martonvásár, Hungary; John Bingham Laboratory, NIAB, Huntingdon Road, Cambridge, CB3 0LE UK

**Keywords:** Genomic prediction, Ridge regression, Metabolomics, Wheat

## Abstract

**Background:**

Genomic prediction of agronomic traits as targets for selection in plant breeding programmes is increasingly common. The methods employed can also be applied to predict traits from other sources of covariates, such as metabolomics. However, prediction combining sets of covariates can be less accurate than using the best of the individual sets.

**Results:**

We describe a method, termed Differentially Penalized Regression (DiPR), which uses standard ridge regression software to combine sets of covariates while applying independent penalties to each. In a dataset of wheat varieties, field traits are better predicted, on average, by seed metabolites than by genetic markers, but DiPR using both sets of predictors is best.

**Conclusion:**

DiPR is a simple and accessible method of using existing software to combine multiple sets of covariates in trait prediction when there are more predictors than observations and the contribution to accuracy from each set differs.

## Background

Increasingly large amounts of data are being collected in crop science experiments. These come from two sources. Firstly, high densities of genetic markers are now available for the major crop groups [1]. Secondly, novel methods are being employed to greatly increase the number of traits that can be captured in any experiment. These may come from specialised phenotypic platforms in controlled environments, field based systems derived from methods of precision agriculture, gene expression experiments, and high throughput analytical platforms such as metabolomics and proteomics [2-4].

A major application for high density genetic markers is to predict traits for selection in breeding programmes [5,6]. These predicted traits can be used in place of direct phenotyping to select among individuals. Since selection is no longer constrained by the time required for phenotyping, rates of response to selection can be greatly increased.

Genetic marker data aside, to date the large amounts of data collected have primarily been from physiological and biochemical analyses of crops rather than for use directly in breeding. An exception is [7] which predicted heterosis for grain yield in maize from 56,110 genetic markers and from 130 seedling metabolites. Both gave good predictions, with the metabolites marginally more accurate. However, there was no report of simultaneous use of both sets of predictors.

In this paper we describe analyses of 151 wheat (*Triticum aestivum var aestivum*) varieties which were studied to determine the extent of diversity in the content and composition of bioactive components [8]. The lines were selected from Europe, Asia, the Americas and Australia and include landraces, breeding lines, modern and older varieties [9]. The analysis of the grain composition of this material has been reported previously [10]. In this paper we use two high throughput analytical systems to determine variation at genomic and metabolite levels and to estimate the relationships among the lines. We show that genetic relationships estimated from the two sources of data are only weakly correlated. Each data source can be used successfully to predict agronomic, yield and grain quality traits measured in field trials, yet pooling the two sources does not commonly give a better result. We therefore develop and test a simple extension to ridge regression to combine information optimally. This method, Differentially Penalized Regression (DiPR), gives predictions which are at least as accurate as predictions from ridge regression on the covariate sets treated alone or in combination.

## Results

### Metabolites

Processing of wholemeal samples of each variety give 620 chemical shift regions for analyses: the “unextracted metabolites”. These represented a mixture of free carbohydrates, organic acids and amino acids, together with signals relating to choline and betaine. Data on a set of 35 more abundant major metabolites was also extracted, including carbohydrates (maltose, sucrose and raffinose) and amino acids (asparagine, glutamate, glutamine, GABA and alanine). For this set, the “extracted metabolites”, the highest fold changes across 151 lines were seen in maltose, fumarate and glutamine (approximately 10 fold). Higher fold changes were observed in trigonelline, tyrosine and tryptophan, although these metabolites were at much lower concentrations in the wheat extracts.

### Genetic distance

Table 1 gives the Mantel statistics (i.e. the correlation) between the distances among varieties estimated from 620 unextracted metabolites, 35 extracted metabolites and 603 genetic markers, and the empirical significance of these correlations. The correlation between extracted and unextracted metabolites is 0.71, but those between marker distances and metabolites are very low, though that for markers and extracted metabolites (0.18) is statistically significant (p = 0.001).

**Table 1:** **Correlation between distance methods**

	**UM**	**EM**
EM	0.708 (10^−5^)	
D	0.062 (0.155)	0.178 (0.001)

D: DArT markers. UM: unextracted metabolites. EM: extracted metabolites. P-value (in brackets) determined by 100,000 permutations.

### Trait prediction

A summary of cross validation correlations (CVCs) between observed and predicted line means for 24 traits is given in Table 2 for predictions based on metabolites alone (extracted and unextracted), DArT markers alone, metabolites and markers combined, and by DiPR in which the two sources of covariates are optimally weighted. DiPR is as good as or better than the best of ridge regression on markers alone, on metabolites alone, or on pooled markers and metabolites for 15 out of 24 comparisons using extracted metabolites and 10 out of 24 comparisons using unextracted metabolites. For 17 out of 24 traits, the maximum correlation is from DiPR on markers with either extracted or unextracted metabolites. For the remaining five traits, the disadvantage of DiPR is slight. Averaged over traits, the correlation for DiPR using extracted metabolites is 0.55 and using unextracted metabolites is 0.52. The next best methods are ridge regression on extracted metabolites alone at 0.51 and the simple combination of unextracted metabolites and markers at 0.49. Treating unextracted and extracted results separately and comparing results from DiPR trait by trait with the best method, the average loss in correlation is 0.0083 for unextracted metabolites and 0.0082 for extracted metabolites. The worst loss, 0.088, is with moisture content for unextracted metabolites. Average losses for the other methods in comparison to the best range from 0.034 (ridge regression on unextracted metabolites and markers combined) to 0.214 (ridge regression on markers alone for extracted metabolites). In summary DiPR is the best on average and when it is not best for an individual trait the loss is usually small.

**Table 2:** **Cross-validation correlations between observed and predicted phenotype**

**Trait**	**D**	**UM**	**EM**	**D + UM**	**D + EM**	**DiPR D + UM**	**DiPR D + EM**	**w DiPR D + UM**	**w DiPR D + EM**
Heading date	0.528	0.568	0.550	**0.594**	0.570	0.574	0.589	0.464	0.251
Plant height	0.609	0.534	0.587	0.615	0.655	0.621	**0.660**	0.688	0.349
Yield	0.234	0.226	0.310	0.301	0.295	**0.315**	0.268	0.699	0.015
Thousand kernel weight	0.465	0.462	0.486	**0.531**	0.481	0.521	0.483	0.453	0.105
Protein	0.221	0.600	**0.666**	0.537	0.378	0.600	**0.666**	*0.000*	*0.000*
Gluten content	0.391	0.581	**0.635**	0.574	0.462	0.558	**0.635**	0.287	*0.000*
Water absorption	0.145	0.412	**0.486**	0.373	0.235	0.400	**0.486**	0.043	*0.000*
Starch content	0.181	0.580	**0.631**	0.549	0.385	0.578	0.620	0.007	0.024
Moisture content	0.095	0.304	0.283	0.312	0.161	**0.323**	0.195	0.353	0.129
Zeleny sedimentation1	0.446	0.590	0.591	0.615	0.515	**0.619**	0.604	0.400	0.111
Hardness Index	0.145	0.384	**0.401**	0.383	0.222	0.379	0.393	0.351	0.048
Test weight	0.648	0.662	0.684	**0.689**	0.689	0.678	0.662	0.456	0.296
Zeleny sedimentation2	0.570	failed	failed	0.492	0.566	**0.570**	0.566	*1.000*	0.996
Protein content1	0.462	0.505	0.538	0.542	0.520	0.522	**0.610**	0.399	0.100
Protein content2	0.323	**0.603**	0.584	0.568	0.456	0.593	0.581	0.101	0.051
kernel weight	0.528	0.391	0.460	0.555	0.545	0.542	**0.563**	0.557	0.250
kernel diameter	0.539	0.440	0.466	0.589	0.565	0.584	**0.607**	0.549	0.202
Hardness Index	0.229	0.540	0.666	0.418	0.263	0.538	**0.688**	0.020	0.050
Moisture content	0.207	0.362	**0.350**	0.304	0.227	0.362	**0.350**	*0.000*	*0.000*
Gluten content	0.163	0.547	**0.586**	0.448	0.307	0.547	**0.586**	*0.000*	*0.000*
Gluten Index	0.449	0.026	0.183	0.379	0.461	0.385	**0.464**	0.790	0.300
Falling Number	0.102	0.783	0.783	0.753	0.627	0.782	**0.788**	0.001	0.152
flour yield	−0.068	0.285	**0.483**	0.152	0.018	0.285	**0.483**	*0.000*	*0.000*
bran yield	0.541	0.360	0.315	0.525	**0.549**	0.544	0.537	0.464	0.251
**Average**	0.340	0.467	0.510	0.492	0.423	0.517	0.545	0.351	0.164
**Maximum**	0.648	0.783	0.783	0.753	0.689	0.782	0.788	1.000	0.996
**Minimum**	−0.068	0.026	0.183	0.152	0.018	0.285	0.195	0.000	0.000

Each of 151 varieties was dropped in turn; a prediction equation computed with drop-one cross-validation from the remaining set of 150 was then used to predict the phenotype of the missing variety. The reported correlations are between 151 observed and predicted phenotypes. D: DArT markers.UM: unextracted metabolites. EM: extracted metabolites. DiPR: differentially penalized regression. w: weighting factor for DiPR. Bold: maximum cross-validation correlation. Italics: w = 0, 0.5 or 1, equivalent to standard ridge regression on metabolites, metabolites + markers, and markers, respectively.

‘w’ is a scaling factor applied to the markers (1-w is applied to the metabolites). The average value used for each trait is listed in the penultimate column of Table 2 for DiPR on markers and unextracted metabolites and in the last column for markers and extracted metabolites. Values of w = 0 and w = 1 occur when DiPR gives identical results to ridge regression on metabolites (w = 0) or on markers (w = 1) alone. These special cases occur for five out of 24 traits predicted from unextracted metabolites and for six traits out of 24 predicted from extracted metabolites. DiPR never gives identical results to simply combining markers and metabolites (w = 0.5). In cases where DiPR is not the best method, its average value of w is commonly close to the value (0, 0.5 or 1) which corresponds to the best method.

DiPR was developed as a better means of combining sets of covariates than simple pooling or merging. The average cross validation correlation from pooling unextracted metabolites and markers is 0.42 in comparison to 0.55 for DiPR. For extracted metabolites the corresponding figures are 0.49 for pooling and 0.52 for DiPR.

## Discussion

Relationships among varieties are similar whether measured by extracted or unextracted metabolites, yet are quite different when measured by markers (Table 1). Nevertheless, both markers and metabolites predict line means reasonably well for most traits (Table 2). The average predictions from markers, unextracted metabolites and extracted metabolites are 0.34, 0.47 and 0.51 respectively: for this data set, better predictions are made by the metabolites. However, for Zeleny sedimentation2 prediction from metabolites fails completely.

One might expect that simply combining the data (w = 0.5) should give better prediction than either set alone. Yet this is the case for only seven and ten out of 24 traits for unextracted and extracted metabolites respectively. The likely explanation is that when one set of covariates has greater predictive power, it may be over-penalized when applying a single penalty to both sets. For this reason, we developed DiPR to apply separate penalties to two sets of covariates. The results in Table 2 demonstrate the advantage of this approach. In some cases simple pooling has performed very poorly in comparison to DiPR, for example with extracted metabolites for water absorption, hardness index, and flour yield.

The 35 extracted metabolite scores are derived from the full spectral dataset of 620 unextracted scores. However, the extracted values generally give higher cross-validation predictions (Table 2). The process of deriving the extracted values to create traits with known biological relevance has therefore also selected against noise in the prediction of the other traits. This serves as a warning that use of large numbers of covariates for prediction should not ignore biological knowledge: a small number of derived traits may perform better than the raw data.

The method we have proposed, DiPR, increases the complexity of analysis by little compared to ridge regression. Consequently, standard ridge regression software can be used. We have searched for the optimum weighting factor for the two sets by passing between the extremes of w = 0 (no weight to markers) and w = 1 (no weight to metabolites) in steps of 0.05. A smaller step size or more sophisticated search strategy would increase accuracy but the improvement in cross-validation correlation would be slight for this dataset. DiPR can also be applied to marker sets in which markers themselves can be classified, for example into polymorphisms in coding and non-coding regions or in candidate and non-candidate genes. DiPR can also be extended to multiple sets of predictors with separate weights for each set. However, although the extension is conceptually simple, a multidimensional search for optimum scaling factors may be prohibitively time consuming without more sophisticated search strategies. Similar approaches to DiPR have also been developed [11,12] to fit mixed models with multiple marker-defined relationship matrices among individuals.

DiPR might also be used to fit the lasso [13] independently to separate sets of covariates; by altering equation 5 to penalize the sum of the absolute values of the regression coefficients rather than the sum of squares.

In the future, we anticipate that high throughput cost effective methods for the various ‘omics disciplines (eg metabolomics, genomics, phenomics) will all be incorporated directly to improve the rate of progress from selection in plant breeding programmes. However, for this to be achieved, these methods must be integrated and focused on target traits of agronomic importance. Though we do not propose our experimental approach for routine use, the analyses we have presented contributes to this goal by showing that two sets of covariates, markers and metabolites, both contain information about diverse agronomic traits and can be combined simply and optimally to give improved prediction of performance of traits of direct interest to the breeder.

The sample set used for this analysis was generated as part of the HEALTHGRAIN project, which aimed to identify lines with increased contents of bioactive components. In most cases these components were not identified in the metabolite profiles compared here, due to their low concentrations or insolubility in the polar aqueous solvent used for extraction. However, the profiles did include some bioactive components, notably betaine and choline [14], which may reduce the risk of cardio-vascular disease by methylation of homocysteine [15,16]. They also contained the amino acid asparagine, which is considered to pose a health risk to consumers due to its conversion to the carcinogen acrylamide during the processing of cereals [17]. Betaine, choline, and asparagine are weakly correlated in this dataset (max correlation choline: asparagine = 0.39). Betaine and choline can be predicted from the DArT markers (cross-validation correlations 0.277 and 0.422 respectively) but asparagine is not (cross-validation correlation 0.073), indicating a low heritability for this metabolite in this dataset.

## Conclusion

DiPR is a simple extension to ridge regression for trait prediction from two or more sets of covariates. We have demonstrated its utility by predicting field and agronomic traits in wheat from genetic marker and seed metabolite data with accuracy close to or better than the best of prediction from markers or seed metabolites alone or from simple concatenation of the two sets. DiPR can be easily implemented using existing software. An R script implementing DiPR is available on request.

## Methods

### Phenotyping

The 151 wheat lines included representatives of all of the major end-use categories, with soft or hard texture, red or white colour and low or high protein content [9]. Data were collected from field trials harvested at the Centre for Agricultural Research the former Agricultural Research Institute of the Hungarian Academy of Sciences, Martonvásár, Hungary in 2005 [18,19]. Traits were measured in the field and the grain were harvested, milled and analysed for additional functional traits as described in [18]. Data for each trait were analysed separately. First line means at each site were estimated in site-by-site analyses taking into account the experimental design. These means were then used as input in subsequent analyses to estimate line means across sites. This gave line means for 24 traits (Table 3) for use in trait prediction. No pair of line means had a correlation squared of >0.95 and all were included in the data analysis.

**Table 3:** **Description of traits**

Heading date	May-June	1	time of flowering in days, where number 1 is the 1th of May
Plant height	cm	2	height of the plants in cm
Yield	kg/plot	3	weight of the seed harvested from a plot
Thousand kernel weight	g/1000kernel	4	weight of 1000 kernels (Hungarian standard MSZ 6367/4-86 (1986))
Protein content	%	5	protein content of the seed estimated by FOSS Tecator 1241, NIR method (ICC Standard No. 202, 159)
Gluten content	%	6	gluten content of the seed estimated by FOSS Tecator 1241, NIR method (ICC Standard No. 202, 159)
Water absorption	%	7	water absorption of the flour estimated by FOSS Tecator 1241, NIR method (ICC Standard No. 202, 159)
Starch content	%	8	starch content of the seed estimated by FOSS Tecator 1241, NIR method (ICC Standard No. 202, 159)
Moisture content	%	9	moisture content of the seed estimated by FOSS Tecator 1241, NIR imethod (ICC Standard No. 202, 159)
Zeleny sedimentation1	ml	10	Zeleny sedimentation estimated by FOSS Tecator 1241, NIR method (ICC Standard No. 202, 159)
Hardness Index		11	hardness of the kernels estimated by FOSS Tecator 1241, NIR method (ICC Standard No. 202, 159)
Test weight	kg/100litre	12	weight of 100 litres of seed measured with FOSS Tecator 1241
Zeleny sedimentation2	ml	13	sedimentation of the flour in lactic acid solution as an estimation of the expected bread volume (ICC Standard No. 116/1)
Protein content1	% flour	14	protein content of the flour measured as n x 5.7 by the Kjeldahl chemical method (ICC105/2)
Protein content2	% wholemeal	15	protein content of the wholemeal measured as N x 5.7 by the Kjedahl chemical method (ICC105/2)
Kernel weight	mg	16	average weight of a kernel measured by Perten SKCS instrument (AACC Method 55–31)
Kernel diameter	mm	17	average diameter of the kernels measured by Perten SKCS instrument (AACC Method 55–31)
Hardness Index		18	average hardness of the seed measured by Perten SKCS instrument (AACC Method 55–31), expressed as an index on a 0–100 scale based on the energy which is required for breakage
Moisture content	%	19	average moisture of the seed measured by Perten SKCS instrument (AACC Method 55–31) based on conductance
Gluten content	%	20	concentration of the gluten protein network formed in the dough determined by washing the starch out of the dough with water during continuous mechanical mixing (ICC137/1)
Gluten Index		21	The gluten index (GI), a measure of dough strength, determined as the gluten remaining on a sieve (g)*100/total gluten (g) (ICC 155) after centrifugation.
Falling Number	s	22	estimate of the α-amylase activity in the flour determined by measuring the falling time of the mixer in the viscoscous solution of flour in a hot water bath. This value relates to the level of α-amylase present as a result of pre-harvest sprouting or pre-maturity amylase production (ICC107/1)
Flour yield	%	23	quantity of white flour produced by milling expressed as a % of the seed weight
Bran yield	%	24	quantity of bran produced by milling as a % of the seed weight

### Genotyping

Whole genome profiling of the lines was carried out using DArT markers by Triticarte Pty Ltd. (www.triticarte.com.au) [20]. Any marker with minor allele frequency (maf) <0.01 was deleted. One of each marker pair with correlation squared >0.95 was deleted, leaving 603 markers out of 843. Markers were scored as 1/0 for presence/absence but rescaled to a mean of zero and a variance of one before analysis. After rescaling, missing marker data were replaced with zero.

### Metabolite profiling

For each wheat line, harvested seed from the field trials was taken and triplicate aliquots of wholemeal (50 mg) extracted at 50°C using 1 mL D2O:CD3OD (80:20) containing d4-TSP (0.05% w/v) as internal standard. The supernatant was heated for 2 minutes at 90°C to remove any residual enzyme activity, before transferring to a 5 mm NMR tube for analysis. NMR spectra were collected at 300°K on an Avance spectrometer (Bruker Biospin, Coventry, UK) equipped with a 5 mm selective inverse probe, operating at 600.0528 MHz. Data were collected using a water suppression pulse sequence with a relaxation delay of 5 s. Each spectrum was acquired using 128 scans of 64 000 data points with a spectral width of 7309.99 Hz. Spectra were automatically Fourier transformed using an exponential window with a line broadening value of 0.5 Hz. Phasing and baseline correction were carried out within the instrument software. 1H chemical shifts were referenced to d4-TSP at δ0.00.

Spectra were automatically referenced to d4-TSP at δ0.00 and reduced using Amix (Analysis of MIXtures software, Bruker Biospin), to ASCII files containing integrated regions or ‘buckets’ of equal width (0.001 ppm). Spectral intensities were scaled to the d4-TSP region (δ0.05 to −0.05). The ASCII file was imported into Excel for the addition of sampling/treatment details.

Signal intensities for characteristic spectral regions for 35 major metabolites were extracted via comparison to library spectra of known standards run under identical conditions. The whole datafiles (“unextracted metabolites”) and data for “extracted metabolites” were used for analysis.

### Genetic distance

Distance matrices among varieties were created separately from DArT data, from unextracted, and from extracted metabolite data using unweighted pair-group averages of Euclidian distances between variety pairs as implemented in the function ‘anges’ in the package ‘cluster’ in R [21]. The significance of correlation between these matrices was tested by Mantel’s method, as implemented in the R package vegan [22] with 10,000 permutations.

### Trait prediction

We wish to compare trait prediction for varieties from markers alone, from metabolites alone and from both sets of predictors simultaneously. With more predictors than observations simple regression methods cannot be used. Ridge regression [23] fits the regression model

1 $$ \mathrm{Y}=\mathrm{X}\mathrm{b} $$

by estimating regression coefficients as

2 $$ \widehat{\mathrm{b}}={\left(\mathrm{X}\hbox{'}\mathrm{X}+\mathrm{I}\uplambda \right)}^{\hbox{-} 1}\mathrm{X}\hbox{'}\mathrm{Y} $$

Y = [y_1_ y_2_ … y_n_]' is a vector of phenotypes for n lines or individuals.

b = [ b_1_b_2_ … b_m_]’ is a vector of m fixed covariate effects.

**X** is the (n x m) design matrix for covariates and assigns values at each covariate to the individual phenotypes in **Y**.

**I** is a unit matrix with the same dimensions as **X’X**.

λ is a positive real number.

The addition of the penalty term **I**λ to **X’X** allows estimates of **b** to be made for all markers simultaneously. If λ is zero, the ridge regression elements reduce to the ordinary least squares solution (which will fail if there are more columns in **X** than rows in **Y**). Estimating **b** from equation 2 is equivalent to estimation in which

3 $$ {\displaystyle {\sum}_1^{\mathrm{n}}{\left({\mathrm{y}}_{\mathrm{i}}\hbox{-} {\displaystyle {\sum}_1^{\mathrm{m}}{\mathrm{b}}_{\mathrm{j}}{\mathrm{x}}_{\mathrm{i}\mathrm{j}}}\right)}^2+\uplambda {\displaystyle {\sum}_1^{\mathrm{m}}{\mathrm{b}}_{\mathrm{j}}^2}} $$

is minimized. Equation 3 has two parts. The first part, ∑(y_i_-∑b_j_x_ij_)^2^, is identical to that minimized in ordinary least squares and corresponds to the squared deviation of observed values (y_i_) from expected (∑b_j_x_ij_). The second part, λ∑b_j_^2^, penalizes the sum of squares of the regression coefficients (b_j_) themselves. The appropriate value of λ can be determined by cross-validation or, for markers, is often set equal to σ_e_^2^/ σ_b_^2^ where σ_e_^2^ and σ_b_^2^ are the residual variance and the variance of the marker effects, respectively [24,25]. In this paper, all estimates of λ come from cross-validation.

We have extended ridge regression to take into account two independent sets of predictors; here metabolites and markers. If markers and metabolites are included together in a standard ridge regression model, both sets will be penalized by a common value of λ. However, if one set has more predictive power it may be over-penalized (shrunk too much) and the other set under-penalized. Similarly, if one set has more covariates than the other it may dominate in estimating λ. Consequently, it is possible that the combined set of predictors will be less accurate than the best set on its own. To avoid this, working on standardized variables, we fit a model in which

4 $$ {\displaystyle {\sum}_1^{\mathrm{n}}{\left({\mathrm{y}}_{\mathrm{i}}\hbox{-} {\displaystyle {\sum}_1^{\mathrm{m}}{\mathrm{b}}_{\mathrm{i}}{\mathrm{x}}_{\mathrm{i}\mathrm{j}}}\hbox{-} {\displaystyle {\sum}_1^{{\mathrm{m}}_{*}}{{\mathrm{b}}^{*}}_{\mathrm{k}}{{\mathrm{x}}^{*}}_{\mathrm{i}\mathrm{k}}}\right)}^2+{\uplambda}_{\mathrm{a}}{\displaystyle {\sum}_1^{\mathrm{m}}{{\mathrm{b}}_{\mathrm{j}}}^2+}{\uplambda}_{\mathrm{b}}{\displaystyle {\sum}_1^{{\mathrm{m}}_{*}}{\mathrm{b}}^{*}{{}_{\mathrm{k}}}^2}} $$

is minimized. The b_j_ and b^*^_k_ are regression coefficients for the two sets of covariates, x and x^*^ with m and m^*^ covariates respectively, each with separate penalties of λ_a_ and λ_b_. The separate penalties ensure that each set of variables is penalized independently. Equation 4 can be reformulated as

5 $$ {\displaystyle {\sum}_1^{\mathrm{n}}{\left({\mathrm{y}}_{\mathrm{i}}\hbox{-} {\displaystyle {\sum}_1^{\mathrm{m}}{\mathrm{b}}_{\mathrm{j}}{\mathrm{x}}_{\mathrm{i}\mathrm{j}}}\hbox{-} {\displaystyle {\sum}_1^{{\mathrm{m}}_{*}}{{\mathrm{b}}^{*}}_{\mathrm{j}}{{\mathrm{x}}^{*}}_{\mathrm{i}\mathrm{k}}}\right)}^2+\uplambda /\mathrm{w}{\displaystyle {\sum}_1^{\mathrm{m}}{{\mathrm{b}}_{\mathrm{j}}}^2+\uplambda /\left(1\hbox{-} \mathrm{w}\right)\;{\displaystyle {\sum}_1^{{\mathrm{m}}_{*}}{\mathrm{b}}^{*}{{}_{\mathrm{k}}}^2}}} $$

In this form, there is a single penalty, λ, for both sets of variables, but with separate weighting factors w and (1-w) where w varies between 0 and 1. The weighting factors can be regarded as additional scaling factors for the standard deviation of the two groups of covariates since a regression on x with an estimated regression coefficient b will give an identical fit to a regression on wx with a regression coefficient b/w. Rescaling by w and 1-w allows fitting of equation 5 by using standard ridge regression software on the rescaled covariates. A search over the 0–1 range of w will then find values of w and λ which maximize the cross-validation correlation between observed and predicted phenotypes. At values of w = 0 and w = 1, equation 5 reduces to ridge regression on a single set of covariates (equation 3). At w = 0.5, both sets of covariates are given equal weight and equation 5 reduces to equation 3 in which no distinction is made between sets of predictors. It follows that, for identical test and training sets, this approach can never give a lower cross-validation correlation than ridge regression on the most accurate set of variables or of ridge regression ignoring the distinction between sets.

As far as we are aware, this form of ridge regression, which for convenience we term Differentially Penalized regression (DiPR) has not been described. In this paper, all models were fitted with the R package Penalized [26] using the following procedure: Each of the 151 varieties was set aside in turn and drop-one cross-validation carried out within the remaining set of 150 varieties. The regression equation from this analysis of 150 varieties was then used to predict the phenotype of the missing variety. The predicted phenotypes of all 151 varieties were collated and correlated with their observed values. Under this procedure the correlation for DiPR is no longer guaranteed to be as good as the best of the other methods: DiPR can fail. An R script is available on request.

### Availability of supporting data

Data used in the analyses are available from http://www.niab.com/pages/id/326/Resources.

## Abbreviations

DiPR: Differentially penalized regression
DArT: Digital array technology
CVC: Cross validation correlation
UM: Unextracted metabolites
EM: Extracted metabolites
D: DArT markers
w: weighting factor for DiPR
